# Case Report: A New Gain-of-Function Mutation of *STAT1* Identified in a Patient With Chronic Mucocutaneous Candidiasis and Rosacea-Like Demodicosis: An Emerging Association

**DOI:** 10.3389/fimmu.2021.760019

**Published:** 2021-12-20

**Authors:** Martin Martinot, Anne Sophie Korganow, Mathieu Wald, Julie Second, Elodie Birckel, Antoine Mahé, Laurent Souply, Mahsa Mohseni-Zadeh, Laure Droy, Julien Tarabeux, Satoshi Okada, Mélanie Migaud, Anne Puel, Aurelien Guffroy

**Affiliations:** ^1^ Infectious Diseases Department, Hôpitaux Civils de Colmar, Colmar, France; ^2^ Department of Clinical Immunology and Internal Medicine, National Reference Center for Systemic Autoimmune Diseases (CNR RESO), Tertiary Center for Primary Immunodeficiency, Strasbourg University Hospital, Strasbourg, France; ^3^ Dermatology Department, Hôpitaux Civils de Colmar, Colmar, France; ^4^ Microbiology Department, Hôpitaux Civils de Colmar, Colmar, France; ^5^ Pathology Department, Hôpitaux Civils de Colmar, Colmar, France; ^6^ Genetic Diagnostic Laboratory, Hôpitaux Universitaires de Strasbourg, Strasbourg, France; ^7^ Laboratory of Human Genetics of Infectious Diseases, Necker Branch, INSERM, UMR 1163, University of Paris, Paris, France; ^8^ Department of Pediatrics, Graduate School of Biomedical and Health Sciences, Hiroshima University, Hiroshima, Japan; ^9^ University of Paris, Imagine Institute, Paris, France; ^10^ St. Giles Laboratory of Human Genetics of Infectious Diseases, The Rockefeller University, New York, NY, United States

**Keywords:** STAT1 GOF, mutation, IL-17, rosacea, Demodex, demodicosis, inborn error of immunity, rosacea-like demodicosis

## Abstract

**Purpose:**

Heterozygous missense *STAT1* mutations leading to a gain of function (GOF) are the most frequent genetic cause of chronic mucocutaneous candidiasis (CMC). We describe the case of a patient presenting a new GOF mutation of *STAT1* with the clinical symptoms of CMC, recurrent pneumonia, and persistent central erythema with papulopustules with ocular involvement related to rosacea-like demodicosis.

**Methods:**

Genetic analysis *via* targeted next-generation sequencing (NGS; NGS panel DIPAI v.1) exploring the 98 genes most frequently involved in primary immunodeficiencies, including *STAT1*, was performed to identify an underlying genetic defect.

**Results:**

NGS identified a novel variant of *STAT1*, c.884C>A (exon 10), p.T295Y, not previously described. This variant was found to be gain of function using an *in vitro* luciferase reporter assay. Rosacea-like demodicosis was confirmed by substantial *Demodex* proliferation observed *via* the microscopic examination of a cutaneous sample. A review of literature retrieved 20 other cases of *STAT1* GOF mutations associated with early-onset rosacea-like demodicosis, most with ocular involvement.

**Conclusion:**

We describe a new *STAT1* GOF mutation associated with a phenotype of CMC and rosacea-like demodicosis. Rosacea-like demodicosis appears as a novel and important clinical phenotype among patients with *STAT1* GOF mutation.

## Introduction

Chronic mucocutaneous candidiasis (CMC) is characterized by increased susceptibility to skin, mucosa, and nail infections caused by *Candida* species and dermatophytes. CMC is found in patients with various acquired or inherited immune disorders (1, 2). The autosomal dominant (AD) signal transducer and activator of transcription protein (STAT) 1 gain of function (STAT1 GOF) is the most frequent genetic cause of CMC (3, 4). STATs are critical signaling molecules downstream of interferons (IFNs), cytokines, growth factors, and hormones, which upon binding to their receptors lead to the activation of Janus kinases, which recruit and phosphorylate cytoplasmic STAT proteins (JAK–STAT signaling pathway). Phosphorylated STATs form homo- or heterodimers and translocate to the nucleus where they bind to specific promoters to initiate transcription (5). Most of the *STAT1* GOF variants are located in the coiled-coil and DNA-binding domains of STAT1 (6). These variants result in enhanced STAT1 phosphorylation, as compared to wild-type *STAT1*, due to impaired nuclear dephosphorylation (4), and enhanced STAT1 signaling downstream of STAT1-dependant cytokines, such as IFN-α/β, IFN-γ, and interleukin (IL)-27, as well as downstream STAT3-dependent cytokines, such as IL-6 and IL-21, resulting in impaired Th17 cell development (4, 7). Patients with *STAT1* GOF present heterogeneous symptoms; CMC is present in nearly all cases often associated with other infectious (bacterial, fungal, or viral) and noninfectious (autoimmunity/inflammatory, aneurysm, and tumor) clinical features (6, 8). Rosacea-like demodicosis is an emerging manifestation reported among the patients with *STAT1* GOF, with only a few cases recently described in the literature (9–14), related to *Demodex* proliferation. We report the case of a patient with a novel heterozygous *STAT1* mutation, shown by functional study to be GOF, who presented CMC associated with recurrent pneumonia and florid rosacea-like demodicosis affecting the center of the face and the eyelids.

## Case Description

A 40-year-old woman has been hospitalized in our department of infectious diseases in February 2018 for fever and cough. Her medical history included CMC since 7 years of age which had never been explored, and esophageal candidiasis in 2015 showing recurrence despite treatment with fluconazole. She had a 3-year-old healthy boy, and no other case was reported in her family.

**Figure 1:**
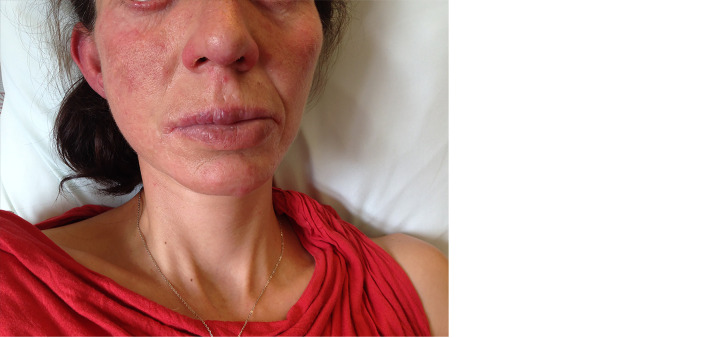
Chronic oral candidiasis (left, photo taken in February 2018) associated with diffuse inflammatory papules and blepharitis along with cutaneous and ocular rosacea-like demodicosis (right, photo taken in June 2018).

Upon admission, the patient was diagnosed with pneumonia associated with bronchiectasis *via* clinical and radiological examinations. Microbiological examination revealed *Streptococcus pneumoniae* in the sputum. On clinical exam, marked oral candidiasis and diffuse inflammatory papules on the face associated with bilateral blepharitis were noted (**Figure 1**). The patient was successfully treated with ceftriaxone but experienced another episode of pneumonia in June 2018, which resolved after further treatment with ceftriaxone. The patient denied receiving any treatment for CMC.

**Figure 2:**
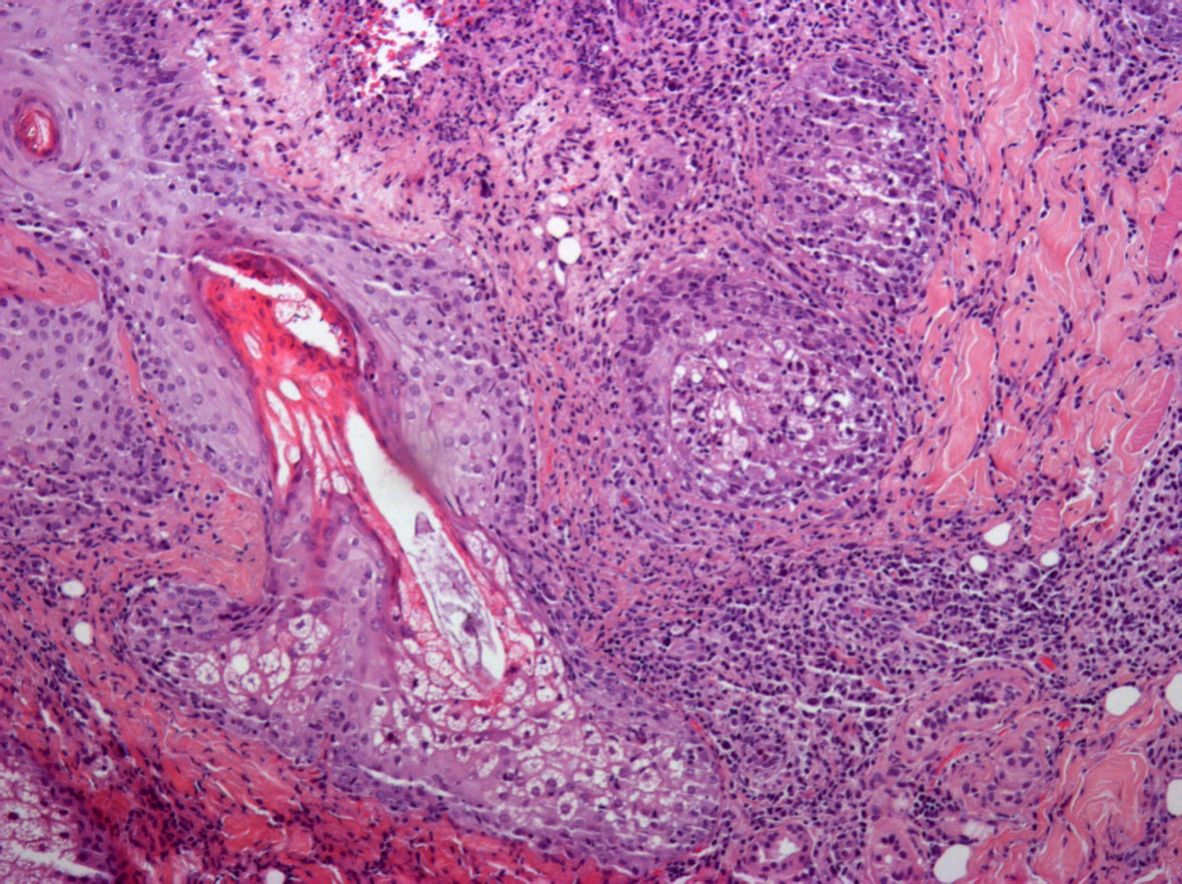
Skin biopsy (hematoxylin and eosin staining, ×10). A mixed inflammatory infiltrate (arrow), without granuloma, related to pilosebaceous units, containing *Demodex* (star).

A direct examination of the eyelashes revealed the presence of *Demodex folliculorum* (**Supplementary Material**, **Appendix 2**). Skin biopsy also revealed non-granulomatous peripilar inflammation with the presence of numerous *Demodex* within the follicles (**Figure 2**). A diagnosis of florid rosacea-like demodicosis was therefore established. Treatment with oral doxycycline (100 mg/day) led to the partial improvement of the lesions. Similarly, oral ivermectin (200 µg/kg, thrice a week) in combination with a daily topical application of ivermectin led to the partial improvement of the lesions. Finally, lasting remission was achieved with the resumption of doxycycline in combination with the local application of 1.5% metronidazole.

## Diagnostic Assessment

### Immune System Evaluation

The absolute lymphocyte count was 1,650/mm^3^, with 1.070/mm^3^ T CD3+ lymphocytes (601 T CD4+, 454 T CD8+, with a CD4/CD8 ratio of 1.32). The natural killer cell count was 166/mm^3^, and the B lymphocyte count was low, with a CD19+ cell count of 58/mm^3^, corresponding to 4% of the total lymphocyte count. Immunoglobulin levels were in the normal range [10.16 g/l (IgG), 2.14 g/L (IgA), and 0.9 g/L (IgM)]. Serologic testing for human immunodeficiency virus was negative. The results of plasma protein electrophoresis for determining immunoglobulin levels (including IgG subclasses) and complement assay were in the normal range, as was the control of tetanus vaccination. The results of autoimmune assay were also negative.

### Next-Generation Sequencing and STAT1 Luciferase Assay

Genetic analysis by targeted next-generation sequencing (NGS; NGS panel DIPAI v.1) exploring the 98 genes most frequently involved in primary immunodeficiencies, including *STAT1*, was performed (**Supplementary Material**, **Appendix 1**). DNA samples were extracted from the peripheral blood. For high-throughput sequencing, targeted libraries were prepared with an individual in-solution SureSelect capture reaction for each DNA sample using a QXT protocol and custom design for genes known to be involved in primary immunodeficiencies (Agilent, Santa Clara, California, USA). Capture experiments were performed using probes corresponding to a panel of 98 genes.

Paired-end sequencing (2 × 75 bp) was performed on Illumina NextSeq 550, multiplexing an average of 25 samples per run. Read mapping, variant calling, and annotation were performed using an in-house bioinformatics pipeline. Detected variants, short indels, and single-nucleotide variants were annotated and ranked using the VaRank software (15).

NGS identified a novel nucleotide change c.884C>A (exon 10) of *STAT1*, at the heterozygous state, resulting in a private missense mutation (p.T295K). The mutation was not confirmed by Sanger sequencing, but we controlled the concordance between NGS data and the individual using TaqMan assay with identitovigilance single-nucleotide polymorphism on an independent sample. Parents’ DNA samples were not available. The mutation was predicted to be deleterious *in silico* (SIFT score = 0.04, CADD score = 20.1) and was not yet described in public databases (1000 Genome Project, gnomAD v2.1.1).

**Figure 3:**
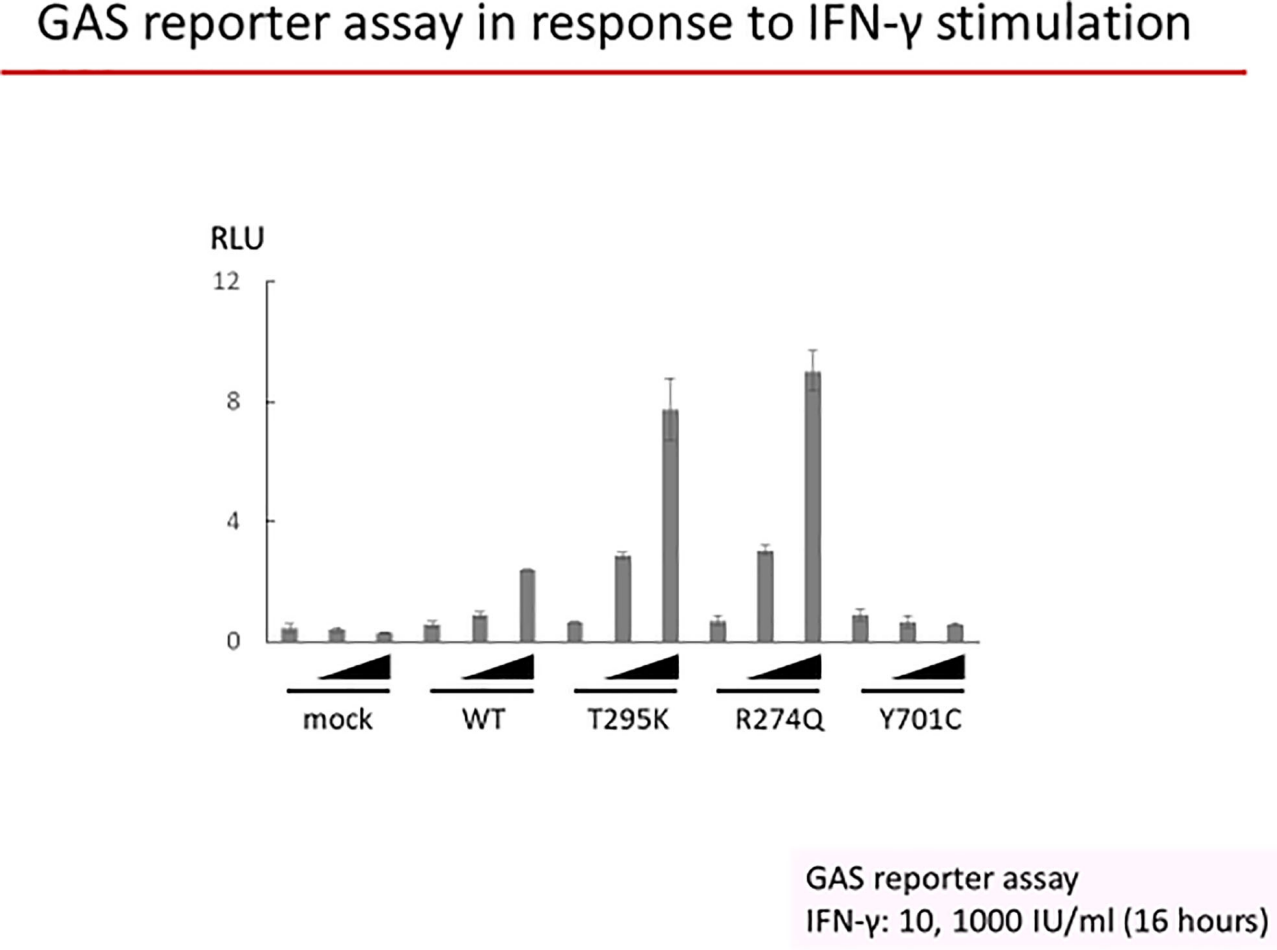
U3C cells were transfected with a mock vector, a WT allele, or three mutant alleles of *STAT1* (encoding T295K, R274Q, or Y701C STAT1). Luciferase activity under a GAS promoter was evaluated after 16 h of stimulation with 10 or 1,000 IU/ml of IFN-γ or without stimulation.

Assessment of the mutation impact was performed *in vitro* using a luciferase reporter assay. U3C cells were plated into 96-well plates (1 × 10^4^/well) and transfected with reporter plasmids (Cignal GAS and ISRE Reporter Assay kit; SA Biosciences) together with plasmids encoding various STAT1 proteins (wild type, WT, or mutant: p.T295K, patient’s mutation, p.R274Q, already reported as GOF, and Y701C, a loss-of-function protein) or an empty vector in the presence of Lipofectamine LTX (Invitrogen, Massachusetts, United States). After 6 h of transfection, cells were washed and incubated in RPMI/10% fetal bovine serum for 24 additional hours. Cells were then stimulated or not with IFN-γ (10 and 1,000 IU/ml) for 16 h, followed by luciferase assay using the Dual-Glo luciferase assay system (Promega, Wisconsin, United States). Experiments were performed in triplicate, and firefly luciferase activity was normalized with Renilla luciferase activity. In this GAS reporter luciferase assay, the patient’s p.T295K-encoding *STAT1* allele showed enhanced luciferase activity upon IFN-γ stimulation, as compared to the WT encoding *STAT1* allele or even more when compared to the loss-of-function p.Y701C-encoding *STAT1* mutant allele, comparable to the p.R274Q-encoding *STAT1* mutant allele, previously shown to be GOF (4, 7) (**Figure 3**). We did not assess pSTAT1 in the patient’s cells.

## Discussion

Inherited CMC has been reported in many inborn errors of immunity impairing the IL-17A/F axis (2, 7). Up to now, the genetic defect responsible for most of the reported cases of CMC is autosomal dominant STAT1 GOF, described in 2011, with various heterozygous mutations located in the coiled-coil domain of *STAT1* associated with exaggerated IFN-α/β and IFN-γ responses and low Th17 cell proportions (3, 4, 7). As many as 105 mutations at 72 amino acid residues, including 65 recurrent mutations, have already been reported in more than 400 patients worldwide (7). *STAT1* GOF can lead to a wide variety of clinical manifestations, with CMC being nearly constantly observed (3, 4, 6, 8). However, rosacea was not reported in the first descriptions of *STAT1* GOF in 2011 (3, 4). A cohort of 26 patients with *STAT1* GOF reported skin infections, such as pustules, furunculosis, or folliculitis, but not rosacea (6). However, some cases of demodicosis can manifest with folliculitis (16). In 2016, a large international cohort of 274 patients with *STAT1* GOF from 167 kindreds originating across 40 countries was described (8). CMC was found in almost all patients (98%); however, the patients displayed a much broader and heterogeneous clinical phenotype, including other skin and invasive infectious diseases, autoimmune diseases (37%), cerebral aneurysm (6%), and/or cancers (6%) (8). Our patient presented CMC, pneumonia, and bronchiectasis, as well as florid rosacea, a clinical form not reported in the two series, but only in 20 other cases among 6 different families (**Table 1**) (9–14). The pathophysiology causing rosacea in *STAT1* GOF remains unknown. However, it is suspected that the immunodeficiency related to *STAT1* GOF facilitates *Demodex* mite and bacterial proliferation involved in rosacea affecting the face and, even more frequently, the eyelids. There is a link between *Demodex* and rosacea (17) explaining the success of ivermectin treatment in some patients with *STAT1* GOF (9, 10). We did not perform a density count for *Demodex*, but the *Demodex* load appeared high and the presence of *Demodex* infection was easily confirmed. However, the antiparasitic treatment was not effective, and it is only in combination with more conventional rosacea therapies that the treatment ultimately achieved the patient’s lasting remission. Other microbes, especially bacteria such as *Bacillus oleronius*, whether they are harbored or not by *Demodex* are involved in the pathophysiology of rosacea. This finding may explain the success of the patient’s treatment with tetracyclines (18). Rosacea is a chronic inflammatory skin disease affecting typically the convexities of the face, with the possibility of ocular involvement (subtype 4) (19). A retrospective study of 115 cases of demodicosis reported three cases related to immunodeficiencies (20). Demodicosis in patients with human immunodeficiency virus infection (21–24), with an immune reconstitution inflammatory syndrome (25–28), or in a patient with ataxia-telangiectasia (29) has been reported. The impaired IL-17-mediated immunity, with low Th17 cell proportions, in STAT1 GOF patients may explain the proliferation of *Demodex* (30), leading to the occurrence of demodicosis, especially rosacea-like demodicosis (31). Rosacea-like demodicosis could also be more prevalent but under-recognized in those with inborn errors of immunity.

**Table 1:** Clinical characteristics of the 19 patients with rosacea and *STAT1* GOF mutation.

Case	Sex	Age (years) Onset/diagnosis	Family	Rosacea	Others clinical manifestations
**1**	F	7/40	1	Facial and ocular	CMC
					Recurrent pneumonia
					Bronchiectasis
**2**	M	5/13	2	Facial and ocular	CMC
					Hypothyroidism
**3**	M	2–3/child	2	Ocular	CMC
					Herpes zoster
					Widespread molluscum contagiosum
**4**	F	Child	2	Facial	Oral and vulvovaginal candidiasis
**5**	F	Adult	2	Facial and Ocular	CMC
					Type 1 diabetes
					Gougerot–Sjögren syndrome
					Coeliac diseases
					B12 and iron deficiency anemia
**6**	F	Birth/14	3	Facial	CMC
**7**	F	6/12	3	Facial and Ocular	Aphthous stomatitis
**8**	F	5	3	Ocular	CMC
**9**	M	Child/44	3	Ocular	CMC
					Pulmonary tuberculosis
					Aphthous stomatitis
**10**	M	7 m/5	4	Facial and ocular	CMC
					Mycobacterial adenitis secondary to BCG
					Recurrent oral herpes
**11**	F	Congenital/23	5	Facial	CMC
					Bilateral hearing loss
					SLE
					Herpes zoster
					Schizophrenia
					Hypothyroidism
**12**	F	20/54	5	Facial	CMC
					Latent type 1 diabetes
					Bronchiectasis
**13**	M	13/52	5	Facial	CMC
					Herpes zoster
					Pernicious anemia
					Intestinal vasculitis
**14**	F	42/50	5	Facial and ocular	CMC
					Pulmonary tuberculosis
**15**	F	16/25	5	Facial	CMC
					Recurrent oral herpes simplex
					Pulmonary tuberculosis
**16**	M	20/85 (deceased)	5	Facial	CMC
					Pulmonary tuberculosis
					Prostatic and tongue cancer
**17**	F	6 m/12	6	Facial	CMC
					Bacterial infections
**18**	F	9/12	7	Facial and ocular	CMC
					Recurrent furunculosis/abscess
					Atopic dermatitis
					Autoimmune cytopenia
					Hypothyroidism (Hashimoto)
**19**	M	15/46	7	Facial	Esophageal candidiasis
					Chronic colitis
**20**	M	7 m/15	7	Facial	CMC
					UTI
					Recurrent aphthous stomatitis
**21**	F	7/7	7	facial	CMC
					UTI
					Recurrent aphthous stomatitis

Family members reference: Family 1 (case report), Family 2 (9), Family 3 (10), Family 4 (11), Family 5 (12), Family 6 (13), and Family 7 (14).

CMC, chronic mucocutaneous candidiasis; SLE, systematic lupus erythematous; BCG, Bacillus Calmette–Guérin vaccine; UTI, urinary tract infection.

In conclusion, we describe a patient heterozygous for a novel *STAT1* GOF mutation. The phenotype includes CMC, bacterial pneumonia, and florid rosacea-like demodicosis with ocular involvement. Rosacea-like demodicosis appears as an increasingly recognized clinical feature among individuals with *STAT1* GOF mutations. Therefore, a thorough cutaneous examination of patients with *STAT1* GOF should carefully evaluate the presence of rosacea-like demodicosis, which can be easily omitted in clinical practice, and rosacea related to chronic demodicosis should be considered among symptoms suggestive of *STAT1* GOF.

## Data Availability Statement

The datasets presented in this article are not readily available because the consent obtained did not include making this publicly available. However, the variant data can be found at https://www.ncbi.nlm.nih.gov/clinvar/ under the accession number SCV001885896. Requests to access the datasets should be directed to the corresponding author.

## Ethics Statement

Written informed consent was obtained from the individual for the publication of any potentially identifiable images or data included in this article.

## Author Contributions

MM, AK, and AG conceived and designed the case report, contributed to the clinical and pathology diagnosis, collected all data, and wrote the manuscript. AP, AK, MM, LS, SO and JT contributed to the pathology diagnosis, immunohistochemistry, and its photographic material. EB, JS, AM, and LD contributed to the dermatologic evaluation and skin biopsies. All authors critically revised the manuscript for important intellectual content, provided approval of the final version, and agreed to be accountable for all aspects of the work. All authors contributed to the article and approved the submitted version.

## Funding

The work was funded by the French National Research Agency (ANR) under the “Investments for the future” program (ANR-10-IAHU-01), the ANR-18-CE93-0008-01, the Integrative Biology of Emerging Infectious Diseases Laboratory of Excellence (ANR-10-LABX-62-IBEID), and the National Institute of Allergy and Infectious Diseases of the NIH (grant no. R01AI127564). This study was supported by the European Reference Networks for Rare Diseases (ERN) Rare Immunodeficiency, Autoinflammatory, and Autoimmune Diseases (RITA).

## Conflict of Interest

The authors declare that the research was conducted in the absence of any commercial or financial relationships that could be construed as a potential conflict of interest.

## Publisher’s Note

All claims expressed in this article are solely those of the authors and do not necessarily represent those of their affiliated organizations, or those of the publisher, the editors and the reviewers. Any product that may be evaluated in this article, or claim that may be made by its manufacturer, is not guaranteed or endorsed by the publisher.
