# Supplementary material for: Case Report: A New Gain-of-Function Mutation of STAT1 Identified in a Patient With Chronic Mucocutaneous Candidiasis and Rosacea-Like Demodicosis: An Emerging Association
Source: Front Immunol. 2021 Dec 20;12:760019. doi: 10.3389/fimmu.2021.760019 (PMC8721043; doi:10.3389/fimmu.2021.760019)
Supplement: Supplementary file 1 [file DataSheet_1.docx]

**Supplementary material appendix 1**

List of 98 genes in DIPAI panel: (annexes)

IL7R; LAT; RAG1; RAG2; DCLRE1C (Artemis); ADA; CD40; CD40L; ICOS; ZAP70; DOCK8; RHOH; TRAC; LCK ; CARD11; RELB; ORAI-I; STAT3; BTK; CD79a; CD79b; BLNK; PIK3CD ; PIK3R1; PTEN; CD19; CD81; CD20; CD21 (CR2); NFKB1; NFKB2; RELA; AICDA; UNG ; PLCG2; FHL1 (COL1A2); HLH PRF1; HLH UNC13D; HLH LYST; HLH RAB27A; HLH FOXP3; IL2RA (CD25); CTLA4; LRBA; FEZF2; NOD2 (CARD15); MST1; TNFRSF6 (FAS); TNFSF6 (FASL); CASP10; CASP8; SH2D1A (XLP1); XIAP; CD27; CD70 (TNFSF7); RASGRP1; MAGT1; PRKDC; CYBB; CYBA; NCF1; NCF2; NCF4; GATA2; CLEC7A; IL12RB1; IL12B; IFNGR1; IFNGR2; STAT1; IRF8; TYK2; RORc; TMC6 (EVER1); TMC8 (EVER2); CXCR4; TLR3; TRAF3IP2 (ACT1); CARD9; IL17RA; IL17RB; IL17F; TMEM173; CECR1 (ADA2) ; C1QA; C1QB; C1QC; C4A; C4B; C2; TLR7; TLR9.

**Supplementary material** Video
